# Retinal microvascular alterations consistent with endothelial dysregulation in paediatric post-COVID-19 syndrome: A prospective matched-cohort study

**DOI:** 10.1038/s41598-026-54086-y

**Published:** 2026-06-03

**Authors:** Pia-Sophie Lamprecht, Lukas Streese, Christoph Hauser, Henner Hanssen, Michael Lorenz, Sascha Klee, Hans Proquitté, Daniel Vilser

**Affiliations:** 1 Department of Paediatrics, Jena University Hospital, Friedrich-Schiller-University, 07747 Jena, Germany; 2 University of Applied Science Niederrhein, 47805 Krefeld, Germany; 3 Department of Nephrology, Medical Faculty, University Hospital Düsseldorf, Heinrich Heine University, 40225 Düsseldorf, Germany; 4 Department of Sport, Exercise and Health, Medical Faculty, University of Basel, Basel- Stadt, 4052 Switzerland; 5 Institute of Biomedical Engineering and Informatics, Technische Universität Ilmenau, 98693 Ilmenau, Germany; 6 Department of General Health Studies, Division Biostatistics and Data Science, Karl Landsteiner University of Health Sciences, Krems an der Donau, 3500 Krems an der Donau, Austria; 7 Clinic for Pediatric and Adolescent Medicine Neuburg/Ingolstadt AMEOS Hospital Association, 86633 Neuburg, Germany

**Keywords:** Post-COVID-19 syndrome, Long COVID in children, Paediatric, Microvascular dysfunction, Endothelial dysregulation, Retinal vessel analysis, Biomarkers, Diseases, Medical research

## Abstract

Persistent symptoms following SARS-CoV-2 infection in children remain poorly understood, and objective biological correlates are scarce. The vascular endothelium is considered a central target of post-viral dysregulation, yet paediatric evidence for microvascular involvement is limited. Retinal imaging enables non-invasive assessment of microvascular structure and function and may help to clarify whether endothelial dysregulation is present in children with post-COVID-19 syndrome (PCS). Retinal vessel diameters and flicker-induced vasoreactivity were assessed at baseline and after a median of 14 weeks. Compared with matched healthy controls, multivariable analyses showed that PCS independently predicted wider central retinal arteriolar equivalent (CRAE + 28.1 μm, 95% CI 21.7–34.5, *p* < 0.001) and central retinal venular equivalent (CRVE + 21.7 μm, 95% CI 15.8–27.7, *p* < 0.001), with a higher arteriolar-to-venular ratio (AVR + 0.038 units, 95% CI 0.012–0.064, *p* = 0.005). These findings suggest a distinct microvascular pattern in children with PCS that is consistent with endothelial dysregulation months after infection. While no significant group-level changes were observed at follow-up, children with particularly large venular diameters and reduced flicker responses showed the greatest improvement. Longer follow-up intervals predicted decreasing venular diameter, and reductions in symptom burden correlated with increasing AVR over time. Together, these results indicate heterogeneous, time-dependent changes in microvascular parameters. Retinal vessel analysis may provide a useful, non-invasive approach to characterising vascular involvement in paediatric PCS and improving understanding of post-viral sequelae.

**Supplementary Information:**

The online version contains supplementary material available at 10.1038/s41598-026-54086-y.

## Introduction

Post-COVID Syndrome (PCS) in children poses a particular challenge because its biological underpinnings remain insufficiently defined, while functional impairment can be substantial and recovery trajectories are highly heterogeneous. Paediatric PCS, also referred to as post-acute infection syndrome (PAIS), describes a constellation of persistent, often fluctuating symptoms that continue beyond the acute phase of SARS-CoV-2 infection and are not attributable to alternative diagnoses^1^.

Although acute COVID-19 is typically mild in paediatric populations, a clinically relevant subgroup experiences prolonged functional impairment, with symptoms including fatigue, exertional intolerance, cognitive complaints, autonomic and orthostatic dysfunction, pain, and reduced participation in daily activities^2^. Rather than representing a single organ disease, PCS is understood as a systemic condition with post-infectious dysregulation across multiple physiological domains^3^. While many children improve over time, others show persistent or relapsing symptoms months after infection^4,5^. In this subgroup, symptoms frequently cluster into recognisable phenotypes, including autonomic, inflammatory, neurocognitive, and exertional intolerance patterns^6–8^. This phenotypic heterogeneity points towards multifactorial pathophysiological processes, rather than a linear sequela of post-viral convalescence.

Reported prevalence estimates in children vary substantially depending on study design, definition criteria, recruitment source, and duration of follow-up^9–11^. Recent population-based data from the UK and the United States suggest an overall prevalence of approximately 1% or lower in children and adolescents^12,13^, consistent with European longitudinal cohorts, including control groups^4,5,14^. Clinic-based cohorts show considerably higher rates among symptomatic, persistently affected patients, reflecting differences in case ascertainment and underrepresentation of more severely affected children in general population cohorts^15^. Symptom severity tends to correlate with functional impairment rather than with the sheer number of symptoms, and children with persistent exertional intolerance or autonomic complaints frequently fall into the more functionally limited subgroup^3^. This heterogeneity underscores the need for objective functional markers that go beyond symptom reporting and enable phenotyping according to biological mechanisms rather than clinical impression alone.

Among the proposed mechanisms underlying PCS— immune dysregulation, viral persistence, autonomic imbalance, microglial activation, mitochondrial dysfunction, thromboinflammation—endothelial alterations have received particular attention^3,16–18^. SARS-CoV-2 has been shown to interact with angiotensin-converting enzyme 2 (ACE2) receptors on the vascular endothelium, facilitating local inflammation, dysregulated vasoregulation, and a state of endothelial activation or dysfunction^19,20^. Rather than structural damage, current evidence supports the concept of functional endothelial dysregulation^20,21^. Several biomarkers support this mechanistic link, including increased von Willebrand factor (vWF) and P-selectin as well as microclots indicating endothelial and platelet activation, elevated adhesion molecules as markers of vascular inflammatory signalling, and alterations in nitric oxide (NO) metabolism reflecting impaired vasodilation^21–26^. While these abnormalities do not occur in all patients, they appear enriched in clinically symptomatic subgroups, particularly those with exertional or autonomic complaints^3^.

Despite increasing mechanistic evidence in adults, paediatric data remain scarce and largely limited to laboratory biomarkers without direct assessment of functional vascular physiology. This represents a key gap in understanding the contribution of microvascular dysregulation in post-COVID conditions in younger populations. The microcirculation is of particular interest because it represents the functional interface between endothelial behaviour and tissue perfusion, and it may help to explain symptoms such as exertional intolerance, orthostatic complaints, and cognitive fatigue that are otherwise difficult to attribute to structural organ pathology.

The retinal vasculature provides a unique, non-invasive window into systemic microvascular function. Retinal vessel analysis has been established as a surrogate of microvascular function and vascular regulation and is commonly used as an indirect marker of function in cardiometabolic and inflammatory conditions^27^. Dynamic assessments of vascular calibre and responsiveness reflect functional endothelial signalling rather than structural remodelling, making them particularly relevant in a condition where functional dysregulation rather than irreversible damage is believed to predominate. Because microvascular physiology reflects early functional changes in vascular regulation, it may provide earlier insights than later structural or organ-level alterations such as tissue damage or clinically apparent organ dysfunction^28,29^.

Given the paucity of paediatric data and the potential role of microvascular and endothelial dysregulation as part of a broader pathophysiological framework in PCS, in vivo assessment of the microcirculation represents a critical next step in characterising post-infectious vascular physiology in affected children. Accordingly, the aim of this study was to investigate retinal microvascular structure and function in children and adolescents with PCS compared with matched healthy peers and to assess their longitudinal evolution.

## Methods

### Study design and participants

We conducted a prospective observational cohort study as part of “Long COVID in Children“ (LongCOCid) project at Jena University Hospital (Germany). Children and adolescents aged 7–17 years with PCS were consecutively recruited from the paediatric Long COVID outpatient clinic at Jena University Hospital between May 2022 and September 2023. Ethical approval was obtained from the Ethics Committee of Jena University Hospital (approval number 2022–2614_3-BO). All methods were performed in accordance with the relevant guidelines and regulations. Written informed consent was obtained from all participants and their legal guardians.

Referrals originated from board-certified paediatricians who suspected post-COVID sequelae based on persistent symptoms after SARS-CoV-2 infection. At the time of recruitment, children and adolescents in Germany underwent routine antigen testing twice weekly, with positive results confirmed by polymerase chain reaction (PCR). Accordingly, the index infection could be dated with high precision in all recruited participants.

PCS was diagnosed according to the guidance of the National Institute for Health and Care Excellence (NICE), which was in effect at the time of study initiation; all patients also met the subsequently published paediatric case definition of the World Health Organization (WHO)^30^, ensuring conceptual consistency with current international diagnostic standards^1^. Verification of prior SARS-CoV-2 infection was based on positive polymerase chain reaction testing in most participants. In the small remaining subgroup, prior infection was supported by positive anti-nucleocapsid SARS-CoV-2 antibodies and clinically compatible infection associated with confirmed household transmission during the acute pandemic phase. Only symptoms newly emerging after infection and persisting beyond the acute phase were considered.

As retinal vessel analysis (RVA) requires sustained attention and cooperation, children younger than seven years were excluded. Patients with ocular conditions precluding reliable RVA, photosensitive epilepsy, or alternative diagnoses explaining symptoms were excluded. For dynamic vessel analysis, both the participants and their custodians had to agree to the application of tropicamide eye drops.

### Assessment of characteristics and symptoms

Height, body mass, heart rate, oxygen saturation, and blood pressure were measured at both study visits. To assess blood pressure, oxygen saturation and heart rate, we used a patient monitoring device (SureSigns VM4, Philips Medical Systems, Hamburg, Germany). Cuff size and positioning followed current paediatric guideline recommendations^31^. All measurements were obtained as a single seated resting measurement prior to static and dynamic vessel analysis. The retinal assessments were conducted as part of a comprehensive, single-day multidisciplinary evaluation programme, including detailed pulmonary, cardiac and neuropaediatric examinations, psychological assessment, and multi-organ ultrasonography.

Symptom burden was evaluated using the Munich Long COVID Symptom Questionnaire (MLCSQ), which captures a total of 96 symptoms across 13 organ system categories. Frequency and severity are rated on a three-point scale, enabling quantitative assessment of symptom load over time. While the questionnaire proved useful for structured symptom assessment in this cohort, comprehensive psychometric validation specifically for paediatric PCS populations is still lacking.

### Static retinal vessel analysis

To ensure high reproducibility and comparability with existing literature^32,33^, static retinal vessel analysis was performed according to the protocols described by Hubbard et al.^34^ and Streese et al.^35^.

The retina was imaged at a 45° angle using a fundus camera (DRS-1, CenterVue SpA, Padova, Italy). After centration of the optic disc and adjustment of brightness, a single photograph was taken; in case of insufficient image quality on the live monitor, the procedure was repeated. The same procedure was subsequently performed in the contralateral eye.

Image analysis was conducted using the Visualis 3.0 software (Visualis Imaging System, Imedos Health GmbH, Jena). After marking the optic disc, the software generated ring-shaped measurement zones at defined distances from the disc margin (Fig. 2A). The measurement area was located 0.5 to 1 disc diameter from the optic disc rim. All arterioles and venules within this zone were semi-automatically detected and labelled. Branching vessels within the zone were included as separate segments. Vessels < 40 μm in diameter or those with unclear vessel classification were flagged but excluded from further analysis. Only photographs with sufficient image quality and full visualisation of the measurement zone were accepted for evaluation.

Based on the Parr–Hubbard formula, the software calculated the central retinal arteriolar equivalent (CRAE), central retinal venular equivalent (CRVE), and the arteriolar-to-venular ratio (AVR)^34^. Because retinal vessel diameters may vary by up to 15% depending on the cardiac cycle timing of image acquisition^36^, the mean value of both eyes was used to increase measurement accuracy.

### Dynamic retinal vessel analysis

Dynamic retinal vessel analysis was performed using the Dynamic Vessel Analyzer (Imedos Systems GmbH, Jena, Germany) to assess flicker light–induced vasodilation. After a short adaptation period, a major temporal arteriole and a corresponding venule within the predefined measurement zone were selected and continuously recorded during baseline. The stimulation protocol consisted of three consecutive cycles, starting with a 50-second baseline recording, followed by 20 s of flicker light stimulation with 12.5 Hz and a recovery phase of 80 s. With a temporal resolution of 25 ms, the system can theoretically capture up to 40 data points per second (≈ 2400 per minute). This protocol is consistent with established methodological recommendations for assessment^37^.

The three cycles were averaged by the system and visualised as a single response curve (Fig. 2B). Vessel segments were selected based on signal stability and recording quality, and only valid curves were accepted for further analysis. The same arteriole and venule were documented and re-evaluated at follow-up to ensure intra-individual comparability.

Mean arteriolar and venular flicker light–induced dilation (aFID and vFID) and arteriolar constriction (aCON) were automatically calculated by the software.

### Control group

SVA data of PCS patients were compared with age-, gender- and body mass index (BMI)-matched healthy controls from the population-based EXAMIN YOUTH cohort (Köchli et al., 2019; Hauser et al., 2023), a separate cohort with minor methodological differences in image acquisition and vessel analysis procedures. Matching was performed on an individual 1:1 basis to minimise confounding by anthropometric factors.

For dynamic vessel analysis, no matched control group or established paediatric reference values are currently available. Therefore, DVA data were interpreted descriptively within the PCS cohort without direct comparison to healthy peers.

### Methodological harmonization with control cohort

Although different fundus camera systems were used (CenterVue DRS-1 versus Topcon TRC NW), both studies applied comparable settings and the same imaging angle (45°). All images were analysed using the identical software. Previous methodological studies have shown that differences between commonly used retinal imaging systems and acquisition protocols have only minor effects on CRAE and CRVE estimates and generally do not result in statistically significant systematic deviations within the relevant measurement range^38–40^.

**Fig. 1:**
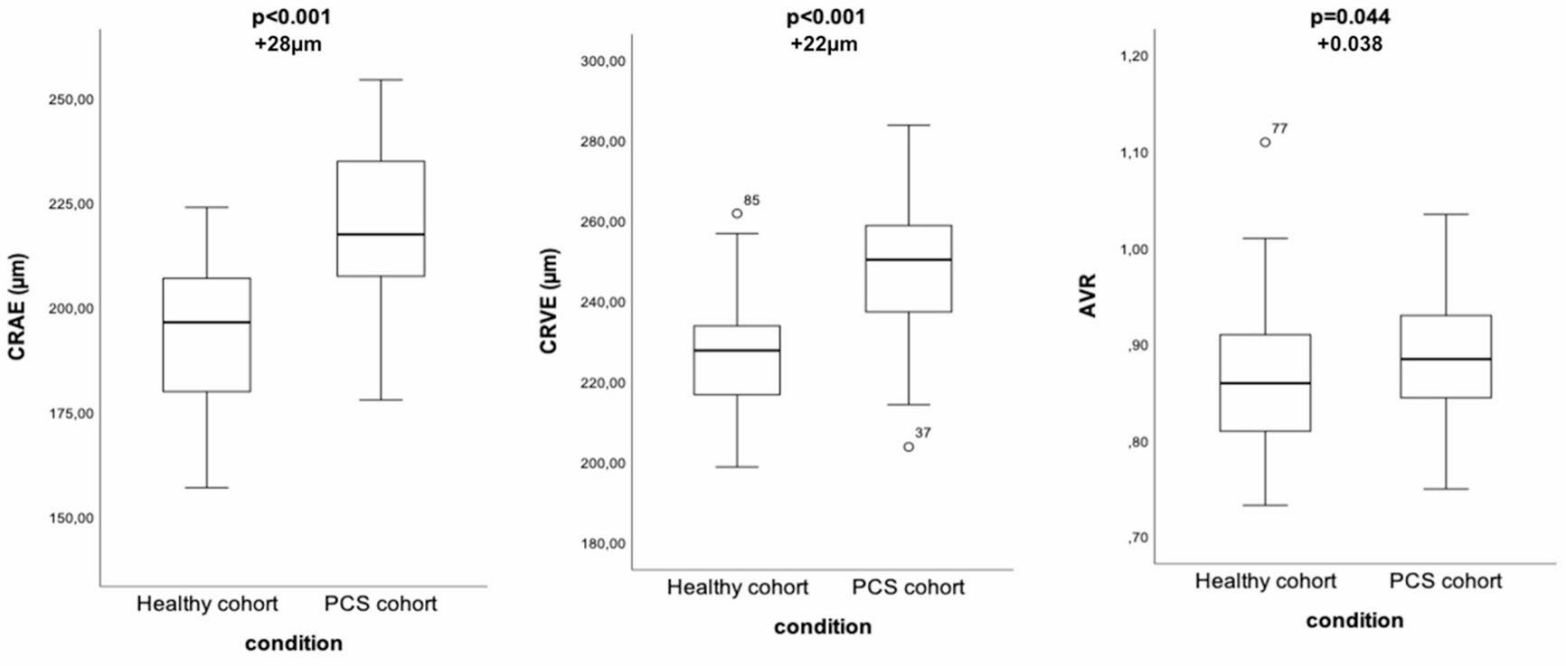
Differences in vascular parameters between patients with PCS and healthy individuals. Each group contains data of 58 children and adolescents. Median values are marked as a horizontal line in the box. All outliers were checked and displayed as a circle. Wilcoxon test for paired samples was used to compare groups. CRAE, central retinal arteriolar equivalent; CRVE, central retinal venular equivalent; AVR, arteriolar-to-venular ratio; PCS, Post-COVID-19 syndrome.

**Fig. 2:**
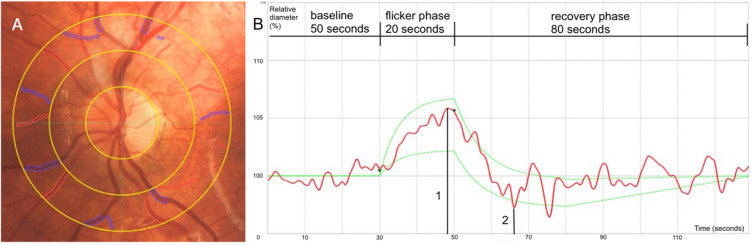
Static and dynamic vessel analysis. **A**: SVA. All arteriolar (red) and venular (blue) segments in the area of 0.5 to 1 papilla diameter away from the margin of the optic disc were marked. **B**: DVA. The red line represents the continuously recorded relative vessel width (%) to the baseline during and after the flicker light phase, calculated as the median of three cycles. The green dashed lines indicate reference boundaries provided by the IMEDOS software, representing the expected range of a physiological, adult flicker response. 1- arteriolar flicker light-induced dilatation (aFID), 2- arteriolar constriction (aCON). (own figure).

While we recorded and analysed one photograph per eye, they recorded two per eye and calculated the mean of all four images for their further analyses. Regarding vessel selection thresholds, both cohorts used cut-offs within the range considered optimal for static retinal vessel analysis (40–80 μm), where diameter-dependent systematic error has been reported to be minimal^40–42^. The difference between the applied thresholds (40 μm versus 45 μm) was therefore considered unlikely to materially affect the comparability of CRAE and CRVE measurements. Visual inspection of the distribution plots demonstrated substantial overlap between cohorts without evidence of marked differences in distributional characteristics or extreme-value structure (Fig. 1, 2). In addition, sensitivity analyses restricted to control participants with retinal vessel diameters within the observed PCS range yielded results comparable to the primary analyses, with persistent significant differences in CRAE, CRVE, and AVR between groups (see Supplementary Table 8). These findings support the robustness of the primary results and argue against a major influence of extreme control values or non-overlapping measurement ranges. Importantly, the magnitude of the observed group differences in our study substantially exceeded the methodological variability reported in the literature, making it unlikely that the findings can be explained solely by acquisition or processing differences. Nevertheless, minor methodological variability cannot be completely excluded.

### Statistical analysis

Statistical analyses were performed using IBM SPSS Statistics (version 29.0.0.0). We used R (version 4.0.2)^43^ to match the Swiss dataset with our own by the MatchIt package (version 4.5.5)^44^. Nearest-neighbour matching without replacement was applied based on age, sex, and BMI. No caliper or distance threshold was specified. The categorical data are given as counts and their percentages, the metric data as median and their corresponding 25th and 75th percentiles. Normality was assessed by visual inspection of histograms and formally tested using the Shapiro-Wilk test. As normal distribution was not met for selected variables, non-parametric tests were applied consistently across analyses. Accordingly, the Wilcoxon signed-rank test (two-tailed) was used for paired samples and the Mann–Whitney U test (two-tailed) for unpaired comparisons. P-values were adjusted for multiple comparisons using the Holm–Bonferroni method.

We compared the SVA parameters (CRAE, CRVE, AVR) and the DVA parameters (aFID, aCON, vFID) between baseline and follow-up, and the SVA parameters between PCS patients and healthy controls.

Spearman correlation analyses were conducted to assess associations with baseline characteristics.

Longitudinal changes were calculated as differences between follow-up and baseline measurements.

Multiple linear regression models were applied to identify determinants of retinal vascular changes between the two examinations. Independent variables included baseline values and the interval between assessments, as well as hospitalisation during acute infection as a marker of disease severity, and the change in MLCSQ scores as an indicator of symptom evolution.

All models were adjusted for age, gender, BMI, and systolic and diastolic blood pressure. Since blood pressure and BMI are known to directly influence retinal vessel diameters and can vary between examination times, the regression analyses included the changes in these measures between baseline and follow-up. Although non-parametric tests were used for group comparisons due to non-normal distributions, linear regression models were considered appropriate as their assumptions were assessed using standard diagnostic plots and were not violated. Results are reported as regression coefficients with the corresponding 95% confidence intervals (CI). A probability of error of 5% (*p* < 0.05) was considered statistically significant.

## Results

### Demographic and clinical data

74 children and adolescents aged 7 to 17 years with PCS were recruited between May 2022 and September 2023 (Table 1). After applying the inclusion and exclusion criteria, 58 static and 59 dynamic retinal vessel analysis recordings (SVA and DVA) met predefined quality criteria. 67 patients (90.5%) returned for follow-up, yielding 48 paired static and 48 paired dynamic datasets for longitudinal evaluation. Symptom burden assessed using the MLCSQ showed no significant change between baseline and follow-up (*p* = 0.554; see Supplementary Table 1). No significant interocular differences in CRAE, CRVE, or the AVR were observed (*p* > 0.05; see Supplementary Table 2). Mean values of both eyes were used for analyses; in a single case, only the left-eye result was included due to insufficient image quality of the right eye.

**Table 1:** Baseline characteristics.

Demographic and clinical characteristics	cohort (*n* = 74)	
	Male	Female
Gender	31 (41.9%)	43 (58.1%)
Age (years)	13 (12;15)	15 (13;16)
Weight (kilogram)	53 (40.2;69)	59 (45;65)
Overweight ^a^	3 (9.7%)	3 (7%)
Adiposity ^a^	2 (6.5%)	0
Systolic blood pressure (mmHg)	111 (107;119)	119 (111;125)
Diastolic blood pressure (mmHg)	68 (62;74)	74 (66;82)
Elevated blood pressure ^b^	5 (16.1%)	14 (32.6%)
Interval between acute SARS-CoV-2 infection and first examination (weeks)	49.7 (35;58,1)	44 (31.4;55.4)
Interval between first and follow-up examination (weeks)	14.1 (13;17.6)	14 (13;16.4)
Score MLCSQ at first examination	12 (9;16)	14 (10;17)
Score MLCSQ at follow-up examination	12.5 (7.3;15)	14 (10;17)
Hospital treatment of acute infection	5 (16.1%)	4 (9.3%)

Categorical data are given as counts (percentages) and metric data are presented as median (interquartile range, 25th–75th percentile). ^a^ Children and adolescents with a weight above the 90th percentile (KIGGS) were categorised as overweight and over the 97th percentile (KIGGS) as obese^74^. ^b^ Children and adolescents with blood pressure values above the 90th percentile (KIGGS) were categorised as having elevated blood pressure^31^.

Retinal vascular parameters were not associated with age or gender (see Supplementary Tables 3 and 4).

### Comparison of retinal microvascular structure between patients with PCS and healthy controls

Matched comparison with healthy peers revealed a distinct retinal microvascular pattern in children and adolescents with PCS (Table 2; Fig. 1). Despite identical age, sex, and BMI distribution, CRAE and CRVE were markedly higher in the PCS cohort, resulting in an increased AVR.

**Table 2:** Characteristics and parameters.

Characteristics and parameters	Healthy cohort (*n* = 58)	PCS cohort (*n* = 58)	*p*-value
Gender (male/female)	23 (39.7%)/35 (60.3%)	24 (41.4%)/34 (58.6%)	
Age (years)	14.9 (12.4;15.5)	14.1 (13.1;16.2)	
Weight (kilogram)	55 (46.2;66.3)	57.8 (44.7;68.5)	
BMI (kg/m^2^)	19.8 (17.5;22.9)	21 (16.8;23.5)	
Systolic blood pressure (mmHg)	112 (104;123)	118 (109;125)	0.067
Diastolic blood pressure (mmHg)	68 (63;73)	72 (63;78)	0.043
CRAE (µm)	197 (180;208)	218 (208;235)	< 0.001
CRVE (µm)	228 (217;235)	251 (237;259)	< 0.001
AVR	0.86 (0.81;0.91)	0.89 (0.84;0.93)	0.044

Categorical data are given as counts (percentages) and metric data are presented as median (interquartile range, 25th–75th percentile). We used the Wilcoxon test for comparison. P-values were adjusted for multiple comparisons using the Holm–Bonferroni method. CRAE, central retinal arteriolar equivalent; CRVE, central retinal venular equivalent; AVR, arteriolar-to-venular ratio; BMI, body mass index.

Although diastolic blood pressure was higher in the PCS group, group differences in retinal vessel parameters remained significant after adjustment for blood pressure (Table 3).

**Table 3:** Association of the Post-COVID-19 syndrome (PCS) with vascular parameters.

Dependent Variable	Regression Models	Independent Variable	Regression coefficient (95%-confidence interval)	*P*-value
CRAE (µm change per unit increase)	Basic	PCS condition	23.595 (17.194;29.995)	< 0.001
	Adjusted	PCS condition	28.089 (21.694;34.483)	< 0.001
		Systolic BP	−0.379 (−0.726;−0.031)	0.033
		Diastolic BP	−0.324 (−0.750;0.102)	0.135
		Hospital treatment	−14.716 (−27.198;−2.234)	0.021
CRVE (µm change per unit increase)	Basic	PCS condition	20.784 (15.242;26.327)	< 0.001
	Adjusted	PCS condition	21.745 (15.794;27.695)	< 0.001
		Systolic BP	−0.205 (−0.528;0.119)	0.213
		Diastolic BP	0.131 (−0.266;0.528)	0.514
		Hospital treatment	−4.881 (−16.496;6.735)	0.407
AVR (units change per unit increase)	Basic	PCS condition	0.023 (−0.002;0.049)	0.072
	Adjusted	PCS condition	0.038 (0.012;0.064)	0.005
		Systolic BP	−0.001 (−0.002;0.001)	0.288
		Diastolic BP	−0.002 (−0.004;0.000)	0.036
		Hospital treatment	−0.041 (−0.092;0.010)	0.111

As we matched for age, gender and body mass index, we did not need to control for those characteristics. We added adjustment for systolic and diastolic blood pressure (BP) and hospital treatment of the acute infection in the following regression models.

CRAE, central retinal arteriolar equivalent; CRVE, central retinal venular equivalent; AVR, arteriolar-to-venular ratio; PCS, Post-COVID-19 syndrome.

In multivariable regression analyses, PCS independently predicted wider arteriolar and venular diameters, with an estimated mean difference of + 28.1 μm for CRAE (95% CI 21.7–34.5, *p* < 0.001) and + 21.7 μm for CRVE (95% CI 15.8–27.7, *p* < 0.001). Correspondingly, AVR was significantly higher in the PCS group (+ 0.038 units, 95% CI 0.012–0.064, *p* = 0.005). Diastolic blood pressure was inversely associated with AVR, and higher systolic blood pressure with narrower CRAE, consistent with known haemodynamic influences in paediatric populations. Hospitalisation status during the acute infection showed no explanatory value for the observed microvascular alterations.

### Changes in SVA and DVA parameters over time in patients with PCS

In our cohort, there was no significant difference in the absolute values of CRAE, CRVE, AVR, aFID, aCON and vFID between baseline and follow-up (all *p* > 0.05; see Supplementary Tables 5 and Supplementary Fig. 1).

### Influence of different parameters on the change in SVA and DVA parameters over time in patients with PCS

#### Value of the individual vascular parameters at initial examination

To explore longitudinal inter-individual variability, multiple linear regression analyses were performed (Table 4).

**Table 4:** Influence of the individual vascular parameters at initial examination on the change over time.

Parameters	Dependent variable	Regression coefficients (95%-confidence interval)	*P*-Value
CRAE at baseline^a^	Change in CRAE between follow-up and baseline^a^	−0.201 (−0.450;0.048)	0.110
CRVE at baseline^a^	Change in CRVE between follow-up and baseline^a^	−0.382 (−0.670;−0.094)	0.011
AVR at baseline^b^	Change in AVR between follow-up and baseline^b^	−0.221 (−0.432;−0.010)	0.040
aFID at baseline^c^	Change in aFID between follow-up and baseline^c^	−0.674 (−1.076;−0.271)	0.002
aCON at baseline^c^	Change in aCON between follow-up and baseline^c^	−0.594 (−0.825;−0.364)	< 0.001
vFID at baseline^c^	Change in vFID between follow-up and baseline^c^	−0.580 (−0.870;−0.290)	< 0.001

The parameters were analysed separately with the dependent variable. Each regression model was adjusted for age, gender, as well as the changes in BMI and systolic and diastolic blood pressure between baseline and follow-up examination.

^a^per 1 μm increase; ^b^per 1unit increase; ^c^per 1% increase.

CRAE, central retinal arteriolar equivalent; CRVE, central retinal venular equivalent; AVR, arteriolar-to-venular ratio; aFID, arteriolar flicker light-induced dilatation; aCON, arteriolar constriction; vFID, venular flicker light-induced dilatation.

Children with wider venules and higher AVR values at the initial examination were more likely to show a decrease in venular diameter over time (–0.382 μm per 1 μm increase in baseline CRVE, 95% CI − 0.670 to − 0.094, *p* = 0.011; − 0.221 units per 1 unit increase in baseline AVR, 95% CI − 0.432 to − 0.010, *p* = 0.040). Lower baseline values of flicker-induced vasoreactivity were associated with later increases in aFID, aCON, and vFID (all *p* < 0.002). These associations indicate heterogeneous intra-individual trajectories in retinal microvascular structure and function over time.

### Influence of hospitalisation at the time of acute infection

We next examined whether hospitalisation during the acute SARS-CoV-2 infection, used as a proxy for disease severity, influenced longitudinal changes in retinal vascular parameters. Regression analyses revealed no significant association between hospitalisation status and changes in SVA or DVA parameters over time (all *p* > 0.05; see Supplementary Table 6).

### Influence of the time interval between first and follow-up examination

We further analysed whether the time interval between baseline and follow-up examination influenced longitudinal changes in retinal vessel parameters. Regression analysis revealed a significant association between follow-up duration and change in venular diameter, with longer intervals associated with a greater reduction in CRVE over time (–1.569 μm per week, 95% CI − 2.829 to − 0.308, *p* = 0.016; Table 5). No statistically significant associations were observed for CRAE, AVR, or dynamic vessel parameters, although a similar trend towards improvement with longer follow-up was noted. These findings indicate that changes in venular calibre may be time-dependent in a subset of children with PCS.

**Table 5:** Influence of the time interval between baseline and follow-up examination on the individual change in vascular parameters over time.

Parameter	Dependent variable	Regression coefficients (95%-confidence interval)	*P*-Value
Interval between baseline and follow-up examination (per 1 week increase)	Change in CRAE between follow-up and baseline^a^	−0.780 (−1.988;0.428)	0.198
	Change in CRVE between follow-up and baseline^a^	−1.569 (−2.829;−0.308)	0.016
	Change in AVR between follow-up and baseline^b^	0.002 (−0.002;0.006)	0.334
	Change in aFID between follow-up and baseline^c^	0.145 (−0.086;0.376)	0.211
	Change in aCON between follow-up and baseline^c^	0.055 (−0.081;0.191)	0.419
	Change in vFID between follow-up and baseline^c^	0.154 (−0.047;0.355)	0.129

The parameters were analysed separately with the dependent variable. Each regression model was adjusted for age, gender, as well as the changes in BMI and systolic and diastolic blood pressure between baseline and follow-up examination.

^a^per 1 μm increase; ^b^per 1unit increase; ^c^per 1% increase.

CRAE, central retinal arteriolar equivalent; CRVE, central retinal venular equivalent; AVR, arteriolar-to-venular ratio; aFID, arteriolar flicker light-induced dilatation; aCON, arteriolar constriction; vFID, venular flicker light-induced dilatation.

### Influence of the change in MLCSQ score

To explore the relationship between clinical symptom evolution and retinal vascular changes, we examined the association between changes in total MLCSQ score and longitudinal differences in SVA and DVA parameters. A significant association was observed for the AVR: children and adolescents who reported a reduction in symptom burden, reflected by a decrease in MLCSQ score, showed a corresponding increase in AVR over time (–0.004 units per one-point increase in MLCSQ, 95% CI − 0.007 to − 0.001, *p* = 0.008; Table 6). No significant associations were identified for CRAE, CRVE, and DVA parameters (all *p* > 0.05). A modest increase in AVR among children reporting symptom improvement should be interpreted cautiously as an exploratory finding that may hint at partial microvascular normalisation during recovery.

**Table 6:** Influence of the change in MLCSQ score on the individual change in vascular parameters over time.

Parameter	Dependent variable	Regression coefficients (95%-confidence interval)	*P*-Value
Change in MLCSQ score (per 1 point increase)	Change in CRAE between follow-up and baseline^a^	−0.341 (−1.347;0.665)	0.496
	Change in CRVE between follow-up and baseline^a^	0.800 (−0.288;1.888)	0.144
	Change in AVR between follow-up and baseline^b^	−0.004 (−0.007;−0.001)	0.008
	Change in aFID between follow-up and baseline^c^	−0.036 (−0.239;0.166)	0.719
	Change in aCON between follow-up and baseline^c^	−0.013 (−0.132;0.106)	0.824
	Change in vFID between follow-up and baseline^c^	0.044 (−0.150;0.237)	0.649

The parameters were analysed separately with the dependent variable. Each regression model was adjusted for age, gender, as well as the changes in BMI and systolic and diastolic blood pressure between baseline and follow-up examination.

^a^per 1 μm increase; ^b^per 1unit increase; ^c^per 1% increase.

CRAE, central retinal arteriolar equivalent; CRVE, central retinal venular equivalent; AVR, arteriolar-to-venular ratio; aFID, arteriolar flicker light-induced dilatation; aCON, arteriolar constriction; vFID, venular flicker light-induced dilatation.

## Discussion

This study provides, to our knowledge, the first in-vivo evidence of retinal microvascular alterations in paediatric PCS obtained through complementary static and dynamic vessel analysis. Compared with matched healthy peers, children with PCS exhibited a coherent pattern of arteriolar and venular dilation with a disproportionate arteriolar contribution reflected in an elevated arteriovenous ratio. These differences persisted after adjustment for blood pressure and acute disease severity and cannot be fully explained by demographic or haemodynamic factors, suggesting that the observed microvascular pattern is associated with PCS and is consistent with endothelial dysregulation. Although specific clinical thresholds for retinal vessel diameters in paediatric PCS are not established, the observed differences exceeded the methodological variability typically reported for retinal vessel analysis systems and were of a magnitude previously associated with cardiovascular risk factors, inflammatory states, and early systemic vascular dysregulation in paediatric and adult cohorts, supporting the potential biological relevance of the present findings.

Our findings may be interpreted within the broader context of recent work indicating biological heterogeneity in paediatric PCS, with temporally structured immune and metabolic trajectories rather than a uniform organ-specific phenotype. In this framework, retinal microvascular alterations may represent one functional correlate of broader systemic dysregulation^7^.

Retinal perfusion is tightly regulated by a coordinated autoregulatory network involving endothelial, myogenic, and neurovascular signalling pathways to sustain the exceptionally high metabolic demands of neural tissue^45,46^. Endothelium-dependent NO bioavailability plays a central role in both basal vascular tone and stimulus-induced vasodilation^47–51^. In the context of SARS-CoV-2 infection, systemic inflammation, endothelial activation, oxidative stress, and dysregulation of the renin–angiotensin system—mediated in part by ACE2 downregulation—have been proposed as mechanisms affecting vascular regulation^52–56^.

Although these mechanisms are most pronounced in severe disease, accumulating evidence demonstrates that endothelial dysregulation can persist long beyond the acute phase^57–59^, potentially resulting in sustained vascular alterations in a subset of individuals^26^.

Sustained low-grade inflammation and microvascular remodelling—particularly capillary rarefication—may impair autoregulatory capacity, leading to passive vessel dilation and reduced vasomotor reserve^60,61^.

These processes are consistent with evidence from adult post-COVID cohorts demonstrating prolonged retinal vessel dilation months after infection. In addition, persistent structural and functional microvascular alterations in PCS, including reduced vascular density—particularly at the capillary level—have been described up to 18 months after infection. These findings are indicative of ongoing endothelial and inflammatory activity observed in post-COVID conditions^60,62–66^.

Our findings extend these observations to the paediatric population. The pattern of vessel dilation in children with PCS—characterised by enlargement of both arterioles and venules with a disproportionate arteriolar contribution—suggests altered vascular regulation rather than fixed structural alteration. In this context, baseline vessel dilation may reflect changes in vascular tone regulation and vasomotor responsiveness, findings that are compatible with endothelial dysregulation described in post-infectious and inflammatory conditions.

Building on these cross-sectional observations, our longitudinal analyses provide insight into the temporal dynamics of these microvascular alterations. While no significant group-level changes were observed over a median follow-up of 14 weeks, substantial inter-individual variability was evident. Children with wider venular diameters at baseline were more likely to show a decrease in venular calibre over time, and higher AVR values at initial examination were associated with a subsequent reduction in AVR.

Furthermore, patients with PCS who exhibited lower baseline values of aFID, aCON, and vFID tended to show greater increases in these parameters, and a longer interval between baseline and follow-up examinations were associated with a greater reduction in venular diameter at follow-up. These findings indicate heterogeneous, time-dependent trajectories of microvascular change related to baseline vascular characteristics, with patterns in some individuals consistent with partial normalisation. While these observations may point towards a relationship between clinical and vascular changes, they should be interpreted with caution, as they may also reflect statistical phenomena related to baseline variability, including regression to the mean, and the observational design limits causal inference.

Hospitalisation during the acute phase of SARS-CoV-2 infection, as a proxy for disease severity, did not predict longitudinal changes in retinal vascular parameters. Laboratory haemostatic, endothelial, and inflammatory parameters, or the symptom burden at the time of acute disease, might have provided a more accurate indication of acute disease severity in paediatric patients; however, such data were not consistently available due to the study design.

Blood pressure is a well-established determinant of retinal vessel calibre. In our SVA measurements, relatively higher systolic blood pressure was associated with narrower arterioles (CRAE), and children with higher diastolic blood pressure exhibited lower AVR values, consistent with previous paediatric studies^47,^^67^. Importantly, the PCS-associated microvascular alterations remained significant after adjustment for blood pressure, indicating that these findings cannot be fully explained by haemodynamic influences alone.

Notably, a relatively high number of children and adolescents with PCS (*n* = 19) showed elevated blood pressure at initial examination. Subgroup analysis revealed reduced CRAE and CRVE in these participants (see Supplementary Table 7), in line with the known influence of blood pressure on retinal vessel calibre. However, these differences did not remain statistically significant after adjustment for multiple comparisons using the Holm–Bonferroni method. All participants underwent a comprehensive cardiovascular evaluation, including clinical examination, electrocardiography, echocardiography, blood pressure assessment, and detailed medical history. Based on the integrated assessment of these findings, no evidence of clinically relevant cardiovascular disease or underlying cardiovascular pathology was identified in any patient. Minor isolated cardiac findings observed in a small number of participants were considered incidental and not explanatory of the PCS phenotype.

In participants presenting with elevated blood pressure values, follow-up evaluations were performed during routine clinical care and were not predefined components of the study protocol. Based on repeated blood pressure measurements and overall cardiological assessment, elevated blood pressure values were not considered clinically significant at the individual patient level. The observation that a higher proportion of patients exhibited elevated blood pressure values therefore only emerged during the subsequent statistical analysis of the cohort. Accordingly, the group-level differences observed in this study should not be interpreted as evidence of a clinically manifest hypertensive or cardiovascular phenotype. Nevertheless, blood pressure values remained modestly elevated on average compared with controls, suggesting that haemodynamic and autonomic influences may still contribute to the observed vascular phenotype. According to the study protocol, retinal vessel analysis results were not incorporated into the clinical evaluations or subsequent management decisions.

Sympathetic nervous system activation, for example in response to stress or fatigue, is known to influence retinal vessel calibres and flicker-light–induced vasodilation^47,^^68^. Following an initial vasoconstrictive phase, there is a subsequent reduction in sympathetic tone, release of NO and metabolic demand may result in a rebound effect characterised by vasodilation. Although efforts were made to ensure a calm and standardised examination environment—including a resting period prior to measurement—complete elimination of stress-related influences is not possible. Considering that fatigue is one of the key features of PCS, even routine procedures may be experienced as more stressful, potentially contributing to elevated blood pressure through increased sympathetic activation^69–71^. Taken together, these findings suggest that elevated blood pressure in this cohort is likely multifactorial and possibly, at least in part, attributable to situational and physiological influences. While a contribution of post-infectious cardiovascular alterations cannot be entirely excluded, the overall pattern argues against a clinically relevant or persistent hypertensive phenotype in this population. Notably, large EHR-based cohort studies have reported an increased occurrence of hypertensive values following SARS-CoV-2 infection in children and adolescents^72^; however, these findings are based on diagnostic coding and do not provide mechanistic insight into underlying vascular processes, highlighting the need for future studies to more precisely characterise blood pressure patterns and their clinical relevance in the post-COVID context. These findings also underscore the methodological importance of adjusting for blood pressure in the statistical analyses. As emphasized above, microvascular alterations remained significant after such adjustment.

This study has limitations that should be considered when interpreting the findings. First, the absence of pre-infection baseline measurements limits the ability to definitively attribute vascular alterations to SARS-CoV-2 infection. Although reference data from a well-characterised cohort of healthy children were used for comparison, minor methodological differences in image acquisition and vessel analysis may have introduced variability, even though the magnitude of group differences in our study makes a major methodological bias unlikely. Second, the relatively long interval between acute infection and initial examination is likely to have influenced the observed findings. As participants were recruited from a specialised Long COVID clinic, they were typically assessed due to persistent symptoms, and systematic evaluations during the early post-infectious phase were not available. Accordingly, the present cohort predominantly reflects a chronic rather than an early post-acute phase of paediatric PCS. While symptom burden generally decreases over time, a substantial proportion of patients continue to report persistent symptoms one year after the acute infection^15,73^. In this context, the persistence of microvascular alterations in our cohort—despite the long interval since infection—is notable. Children and adolescents with such a prolonged symptom history may require extended follow-up periods to demonstrate more pronounced or consistent changes. This assumption is supported by our finding that patients with a longer interval between examinations were more likely to show a decrease in CRVE. In a small number of participants, prior SARS-CoV-2 infection could not be confirmed by PCR testing and was instead inferred from serological findings together with clinically compatible infection in the context of confirmed household transmission, which may have introduced limited potential for misclassification.

Third, participants were recruited from a specialised paediatric Long COVID clinic, which may have led to an overrepresentation of individuals with more persistent or severe symptoms. This potential selection bias may limit the generalisability of the findings to the broader population of children and adolescents following SARS-CoV-2 infection. Fourth, while DVA provides dynamic measures of retinal vascular function and is commonly used as a surrogate for neurovascular coupling, the absence of established paediatric normative values limits the interpretation of functional measures.

Fifth, although useful for structured longitudinal symptom assessment, the MLCSQ has not yet undergone comprehensive psychometric validation specifically in paediatric PCS populations.

Finally, as discussed above, residual confounding by blood pressure, stress, or autonomic activation cannot be fully excluded despite statistical adjustment and standardised examination procedures. In addition, blood pressure assessment was based on single seated resting measurements obtained within a complex multidisciplinary clinical evaluation rather than repeated measurements according to paediatric hypertension guidelines, which may have introduced additional measurement variability.

Despite these limitations, the consistency of the structural and functional alterations observed across multiple vascular parameters supports the robustness of the main conclusions.

This study provides the first in-vivo evidence of altered retinal microvascular parameters in children and adolescents with PCS. Compared with healthy peers, affected individuals showed significantly enlarged arteriolar and venular diameters, suggesting persistent alterations in microvascular regulation several months after SARS-CoV-2 infection. Longitudinal analyses indicated heterogeneous, time-dependent changes in vascular parameters, with individuals showing varying trajectories related to baseline vascular characteristics. While some patterns were consistent with partial normalisation over time, these findings should be interpreted with caution given the observational design.

In light of the fundamental role of the microcirculation, including endothelial function, in regulating tissue perfusion, these findings offer important insights into the biological underpinnings of prolonged symptoms in paediatric PCS. Retinal imaging may represent a useful, non-invasive approach for assessing microvascular involvement in this context. Further longitudinal studies are required to validate these findings, to better characterise temporal patterns, and to determine whether retinal vascular parameters may serve as markers of disease course in children and adolescents with PCS.

## Electronic Supplementary Material

Below is the link to the electronic supplementary material.

Supplementary Material 1

## Data Availability

Deidentified individual participant data (including data dictionary) will be made available upon reasonable request to the corresponding author (daniel.vilser@ameos.de) beginning 3 months and ending 5 years after publication, for researchers who provide a methodologically sound proposal that is approved by the institutional ethics committee. Data will be shared via secure file transfer after signing a data access agreement.

## Declarations

### Data sharing statement

Deidentified individual participant data (including data dictionary) will be made available upon reasonable request to the corresponding author (daniel.vilser@ameos.de) beginning 3 months and ending 5 years after publication, for researchers who provide a methodologically sound proposal that is approved by the institutional ethics committee. Data will be shared via secure file transfer after signing a data access agreement.
