# Supplementary material for: Retinal microvascular alterations consistent with endothelial dysregulation in paediatric post-COVID-19 syndrome: A prospective matched-cohort study
Source: Sci Rep. 2026 Jun 3;16:17180. doi: 10.1038/s41598-026-54086-y (PMC13234433; doi:10.1038/s41598-026-54086-y)
Supplement: Supplementary file 1 — Supplementary Material 1 [file 41598_2026_54086_MOESM1_ESM.docx]

Retinal microvascular alterations consistent with endothelial dysregulation in paediatric post-COVID-19 syndrome: a prospective matched-cohort study

Pia-Sophie Lamprecht, Lukas Streese, Christoph Hauser, Henner Hanssen, Michael Lorenz, Sascha Klee, Hans Proquitté, Daniel Vilser^*^

# Supplementary data

**Supplementary Table 1** Comparison of MLCSQ score between both points in time using the Wilcoxon test for paired samples.

| **Parameter** | **Initial examination (n=74)** | | | **Follow-Up examination (n=67)** | | | **p-value** |
| --- | --- | --- | --- | --- | --- | --- | --- |
|  | Median | First quartile | Third quartile | Median | First quartile | Third quartile |  |
| MLCSQ score | 13.50 | 10.00 | 16.25 | 13.00 | 9.00 | 15.00 | 0.554 |

MLCSQ, Munich Long Covid Symptom Questionnaire.

**Supplementary Table 2** Comparison of CRVE, CRAE and AVR between both eyes using the Wilcoxon test for paired samples.

| **Point in Time** | **Parameters** | **Right Eye (n=57)** | | | **Left Eye (n=47)** | | | **p-value** |
| --- | --- | --- | --- | --- | --- | --- | --- | --- |
|  |  | Median | First quartile | Third quartile | Median | First quartile | Third quartile |  |
| Initial Exami  nation | CRVE (µm) | 250 | 235 | 260 | 250 | 238 | 261 | 0.629 |
|  | CRAE (µm) | 220 | 205 | 237 | 220 | 208 | 230 | 0.796 |
|  | AVR | 0.89 | 0.84 | 0.94 | 0.87 | 0.83 | 0.92 | 0.310 |
| Follow-Up Exami  nation | CRVE (µm) | 250 | 241 | 259 | 250 | 240 | 262 | 0.353 |
|  | CRAE (µm) | 224 | 216 | 231 | 226 | 208 | 238 | 0.580 |
|  | AVR | 0.89 | 0.86 | 0.94 | 0.88 | 0.83 | 0.93 | 0.244 |

AVR, arteriolar-to-venular diameter ratio; CRAE, central retinal arteriolar equivalent; CRVE, central retinal venular equivalent.

**Supplementary Table 3** Distribution of SVA and DVA parameters at both points in time separated for the different genders.

| **Point in Time** | **Parameters** | **Male** | | | **Female** | | | **p-value** |
| --- | --- | --- | --- | --- | --- | --- | --- | --- |
|  |  | Median | First quartile | Third quartile | Median | First quartile | Third quartile |  |
| Initial Exami  nation | CRVE (µm) | 250 | 238 | 264 | 251 | 235 | 259 | 0.554 |
|  | CRAE (µm) | 217 | 202 | 236 | 218 | 209 | 234 | 0.542 |
|  | AVR | 0.89 | 0.84 | 0.92 | 0.88 | 0.85 | 0.93 | 0.794 |
|  | aFID (%) | 3.42 | 2.23 | 5.28 | 2.77 | 1.79 | 4.30 | 0.472 |
|  | aCON (%) | -4.08 | -4.67 | -2.85 | -3.62 | -4.68 | -1.59 | 0.647 |
|  | vFID (%) | 4.13 | 3.03 | 6.78 | 4.29 | 2.67 | 5.88 | 0.528 |
| Follow-up exami  nation | CRVE (µm) | 249 | 242 | 261 | 249 | 235 | 262 | 0.836 |
|  | CRAE (µm) | 223 | 213 | 233 | 224 | 216 | 236 | 0.918 |
|  | AVR | 0.87 | 0.83 | 0.93 | 0.89 | 0.86 | 0.93 | 0.887 |
|  | aFID (%) | 3.68 | 1.85 | 4.49 | 2.58 | 1.33 | 3.40 | 0.877 |
|  | aCON (%) | -3.80 | -4.81 | -2.72 | -2.92 | -4.57 | -2.00 | 0.234 |
|  | vFID (%) | 3.59 | 2.92 | 6.69 | 3.56 | 2.11 | 5.70 | 0.535 |

AVR, arteriolar-to-venular diameter ratio; CRAE, central retinal arteriolar equivalent; CRVE, central retinal venular equivalent; aFID, arteriolar flicker light-induced dilation response; aCON, arteriolar constriction; vFID, venular flicker light-induced dilation.

**Supplementary Table 4** Spearman’s Correlations between Age or Gender and the different RVA parameters.

| **Parameters** | **Age (years)** | | **Gender (male)** | |
| --- | --- | --- | --- | --- |
|  | Correlation Coefficient | p-value | Correlation Coefficient | p-value |
| CRVE (µm) | 0.075 | 0.575 | 0.026 | 0.846 |
| CRAE (µm) | -0.055 | 0.683 | -0.043 | 0.749 |
| AVR | -0.158 | 0.236 | -0.052 | 0.696 |
| aFID (%) | -0.012 | 0.926 | 0.168 | 0.202 |
| aCON (%) | 0.071 | 0.592 | -0.096 | 0.468 |
| vFID (%) | -0.019 | 0.886 | 0.076 | 0.566 |

A probability of error of 5% (p < 0.05) was considered as statistically significant.

AVR, arteriolar-to-venular diameter ratio; CRAE, central retinal arteriolar equivalent; CRVE, central retinal venular equivalent; aFID, arteriolar flicker light-induced dilation response; aCON, arteriolar constriction; vFID, venular flicker light-induced dilation.

**Supplementary Table 5** Comparison of SVA and DVA parameters at both points in time

| **Parameters** | **First examination**  **(SVA n=58, DVA n=59)** | | | **Follow-up examination**  **(SVA n=48, DVA n=48)** | | | **p-value** |
| --- | --- | --- | --- | --- | --- | --- | --- |
|  | Median | First quartile | Third quartile | Median | First quartile | Third quartile |  |
| CRAE (µm) | 218 | 208 | 235 | 224 | 213 | 236 | 0.969 |
| CRVE (µm) | 251 | 237 | 259 | 249 | 241 | 260 | 0.271 |
| AVR | 0.89 | 0.84 | 0.93 | 0.88 | 0.85 | 0.93 | 0.496 |
| aFID (%) | 3.03 | 2.14 | 4.74 | 2.73 | 1.68 | 4.02 | 0.369 |
| aCON (%) | -3.79 | -4.64 | -2.24 | -3.23 | -4.68 | -2.33 | 0.852 |
| vFID (%) | 4.28 | 2.97 | 5.97 | 3.58 | 2.37 | 5.81 | 0.907 |

We used the Wilcoxon test for paired samples for comparison of the individual parameters.

SVA, static retinal vessel analysis; DVA, dynamic retinal vessel analysis; CRAE, central retinal arteriolar equivalent; CRVE, central retinal venular equivalent; AVR, arteriolar-to-venular ratio; aFID, arteriolar flicker light-induced dilatation; aCON, arteriolar constriction; vFID, venular flicker light-induced dilatation.

**Supplementary Figure 1** Visualisation of the results of SVA (A, B and C) and DVA (D, E and F) at initial and follow-up examination. For SVA parameters boxplots represents data of n=58 children and adolescents at first examination and n=48 at follow-up examination. For DVA parameters there is a number of n=59 at initial examination and n=48 at follow-up examination. Median values are marked as a vertical line in the box. All outliers were checked and displayed as a dot. Wilcoxon test for paired samples was used to compare groups. SVA, static retinal vessel analysis; DVA, dynamic retinal vessel analysis; CRAE, central retinal arteriolar equivalent; CRVE, central retinal venular equivalent; AVR, arteriolar-to-venular ratio; aFID, arteriolar flicker light-induced dilatation; aCON, arteriolar constriction; vFID, venular flicker light-induced dilatation.


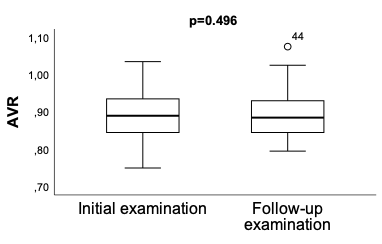

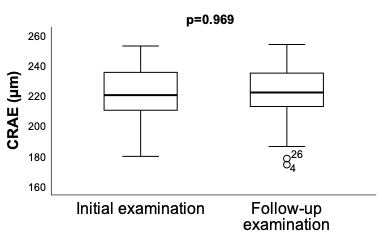

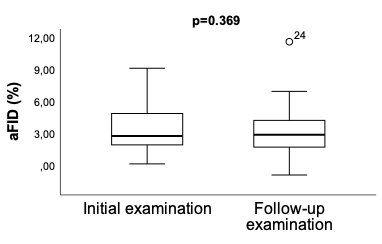

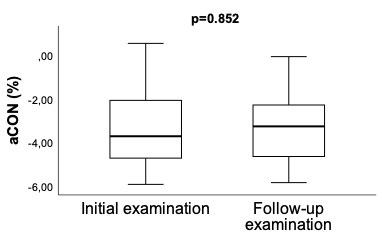

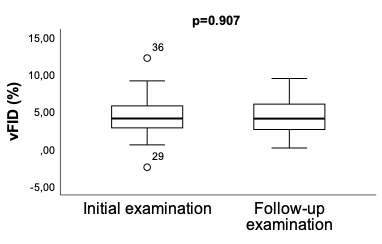

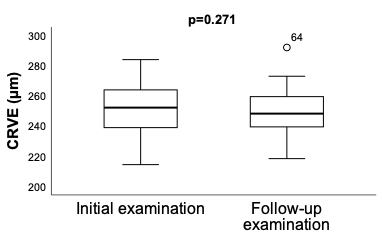


A

B

C

D

E

F

**Supplementary Table 6** Influence of hospitalisation on the individual change in vascular parameters over time

| **Parameter** | **Dependent variable** | **Regression coefficients**  **(95%-confidence interval)** | **P-Value** |
| --- | --- | --- | --- |
| Hospitalisation at the time of acute infection | Change in CRAE between follow-up and baseline^a^ | -1.277 (-22.059;19.505) | 0.901 |
|  | Change in CRVE between follow-up and baseline^a^ | -3.617 (-26.616;19.382) | 0.751 |
|  | Change in AVR between follow-up and baseline^b^ | 0.002 (-0.066;0.069) | 0.958 |
|  | Change in aFID between follow-up and baseline^c^ | -0.115 (-3.458;3.228) | 0.945 |
|  | Change in aCON between follow-up and baseline^c^ | -0.834 (-2.775;1.108) | 0.390 |
|  | Change in vFID between follow-up and baseline^c^ | -2.362 (-5.460;0.737) | 0.131 |

The parameters were analysed separately with the dependent variable. Each regression model was adjusted for age, gender, as well as the changes in BMI and systolic and diastolic blood pressure between baseline and follow-up examination.

^a^per 1µm increase; ^b^per 1unit increase; ^c^per 1% increase

CRAE, central retinal arteriolar equivalent; CRVE, central retinal venular equivalent; AVR, arteriolar-to-venular ratio; aFID, arteriolar flicker light-induced dilatation; aCON, arteriolar constriction; vFID, venular flicker light-induced dilatation.

**Supplementary Table 7** Comparison of RVA parameters based on blood pressure

| **Parameters** | **Normal blood pressure at initial examination (n=55)** | | | **Elevated blood pressure at initial examination (n=19)** | | | **p-value** | **Adjusted p-value** |
| --- | --- | --- | --- | --- | --- | --- | --- | --- |
|  | Median | First quartile | Third quartile | Median | First quartile | Third quartile |  |  |
| CRAE (µm) | 223 | 210 | 236 | 211 | 200 | 219 | 0.015 | 0.075 |
| CRVE (µm) | 254 | 240 | 264 | 238 | 232 | 250 | 0.009 | 0.054 |
| AVR | 0.90 | 0.84 | 0.94 | 0.87 | 0.85 | 0.92 | 0.244 | 0.976 |
| aFID (%) | 2.70 | 2.04 | 4.81 | 3.70 | 2.55 | 4.92 | 0.384 | >0.999 |
| aCON (%) | -3.77 | -4.67 | -1.75 | -3.96 | -4.67 | -2.56 | 0.738 | >0.999 |
| vFID (%) | 3.91 | 2.77 | 6.74 | 4.74 | 3.38 | 5.74 | 0.604 | >0.999 |

We used the Mann-Whitney U test for comparison. Children and adolescents with a blood pressure over the 90th percentile (KIGGS) were categorised as having elevated blood pressure. CRVE and CRAE were found to be lower in cases of elevated blood pressure (p<0.05). These differences did not remain statistically significant after adjustment for multiple comparisons using the Holm–Bonferroni method.

AVR, arteriolar-to-venular diameter ratio; CRAE, central retinal arteriolar equivalent; CRVE, central retinal venular equivalent; aFID, arteriolar flicker light-induced dilation response; aCON, arteriolar constriction; vFID, venular flicker light-induced dilation.

**Supplementary Table 8** Comparison of SVA parameters after sensitivity analysis

| **Characteristics and parameters** | **Healthy cohort** | **PCS cohort** | **p-value** |
| --- | --- | --- | --- |
| CRAE (µm) | 197 (182;207) | 218 (208;235) | <0.001 |
| CRVE (µm) | 229 (218;236) | 251 (237;259) | <0.001 |
| AVR | 0.86 (0.81;0.91) | 0.89 (0.84;0.93) | 0.038 |

Sensitivity analyses restricted to control participants with retinal vessel diameters within the observed PCS range yielded comparable effect patterns, supporting the robustness of the primary findings.

AVR, arteriolar-to-venular diameter ratio; CRAE, central retinal arteriolar equivalent; CRVE, central retinal venular equivalent
